# GZ17-6.02 kills prostate cancer cells *in vitro* and *in vivo*

**DOI:** 10.3389/fonc.2022.1045459

**Published:** 2022-11-03

**Authors:** Laurence Booth, Jane L. Roberts, Cameron West, Paul Dent

**Affiliations:** ^1^ Department of Biochemistry and Molecular Biology, Virginia Commonwealth University, Richmond, VA, United States; ^2^ Genzada Pharmaceuticals, Sterling, KS, United States

**Keywords:** GZ17-6.02, olaparib, PARP1, autophagy, ER stress, ATM

## Abstract

GZ17-6.02 is undergoing clinical evaluation in solid tumors and lymphoma. We defined the biology of GZ17-6.02 in prostate cancer cells and determined whether it interacted with the PARP1 inhibitor olaparib to enhance tumor cell killing. GZ17-6.02 interacted in a greater than additive fashion with olaparib to kill prostate cancer cells, regardless of androgen receptor expression or loss of PTEN function. Mechanistically, GZ17-6.02 initially caused peri-nuclear activation of ataxia-telangiectasia mutated (ATM) that was followed after several hours by activation of nuclear ATM, and which at this time point was associated with increased levels of DNA damage. Directly downstream of ATM, GZ17-6.02 and olaparib cooperated to activate the AMP-dependent protein kinase (AMPK) which then activated the kinase ULK1, resulting in autophagosome formation that was followed by autophagic flux. Knock down of ATM, AMPKα or the autophagy-regulatory proteins Beclin1 or ATG5 significantly reduced tumor cell killing. GZ17-6.02 and olaparib cooperated to activate protein kinase R which phosphorylated and inactivated eIF2α, i.e., enhanced endoplasmic reticulum (ER) stress signaling. Knock down of eIF2α also significantly reduced autophagosome formation and tumor cell killing. We conclude that GZ17-6.02 and olaparib interact to kill prostate cancer cells *in vitro* by increasing autophagy and by enhancing ER stress signaling. *In vivo*, GZ17-6.02 as a single agent profoundly reduced tumor growth and significantly prolonged animal survival. GZ17-6.02 interacted with olaparib to further suppress the growth of LNCaP tumors without ultimately enhancing animal survival. Our data support the consideration of GZ17-6.02 as a possible therapeutic agent in patients with AR+ prostate cancer.

## Introduction

GZ17-6.02 has three components: curcumin, harmine and isovanillin and is presently undergoing phase I safety evaluation in cancer patients with solid tumors and lymphoma (NCT03775525) (1–6). Over the past two years we have published data demonstrating that GZ17-6.02 kills a wide range of tumor cell types, including ER+ breast, colorectal, pancreatic, hepatic, biliary, NSCLC, cutaneous melanoma, sarcoma and actinic keratoses (1–6).

Our prior data using GZ17-6.02 demonstrated that it activated a DNA damage (ATM) and metabolism regulatory (AMPK) pathway, which resulted in enhanced autophagosome formation that was followed by autolysosome formation (autophagic flux). GZ17-6.02 activated PKR-like endoplasmic reticulum kinase (PERK) and increased the phosphorylation (inactivation) at serine 51 of eIF2α, with ER stress signaling facilitating the autophagy response. The mechanisms of cell killing were multi-faceted requiring autophagy, ER stress signaling and death receptor signaling (1–6).

Prostate cancer, after cutaneous melanoma, is a major cause of cancer in US males (7). Successful treatment of the disease localized to the prostate utilizes surgery, brachytherapy, external beam radiotherapy and therapeutic interventions including anti-androgens and cytotoxic drugs such as Taxanes (8, 9). Non-organ-confined prostate cancer is usually treated first by androgen deprivation therapy (surgical or chemical castration) and then by AR signaling inhibitors. Treatment with Taxanes is used once resistance to full androgen blockade has been observed. In patients with disease localized in the prostate, their 5-year survival nears 100%, and even in patients who present with disseminated disease, a third will survive for at least 5 years. The PARP1 inhibitors olaparib and rucaparib are FDA approved for prostate cancer patients who present with advanced castration-resistant disease with homologous recombination repair deficits. For olaparib, recent clinical data strongly argues that the tumors of patients who have loss of function in BRCA1 and/or BRCA2 are most responsive to PARP1 inhibitors (10). The prostate cancer cell lines DU145, PC3 and LNCaP are commonly used for *in vitro* studies of the disease; DU145 and LNCaP have mutations in BRCA1 and BRCA2 whereas PC3 does not. The present studies were designed to investigate the biology of GZ17-6.02 in prostate cancer cells, and to define its interaction with the FDA-approved Poly ADP-ribosyl Polymerase 1 (PARP1) therapeutic olaparib.

## Materials and methods

### Materials

The LNCaP, PC3 and DU145 cell lines were obtained from the ATCC (Bethesda, MD). Olaparib was purchased from Selleckchem (Houston, TX). All Materials were obtained as described in the references (1–6). Trypsin-EDTA, DMEM, RPMI, penicillin-streptomycin were purchased from GIBCOBRL (GIBCOBRL Life Technologies, Grand Island, NY). Other reagents and performance of experimental procedures were as described (1–6). Antibodies were purchased from Cell Signaling Technology (Danvers, MA); Abgent (San Diego, CA); Novus Biologicals (Centennial, CO); Abcam (Cambridge, UK); and Santa Cruz Biotechnology (Dallas, TX). Cell Signalling antibodies: ATM (D2E2) Rabbit mAb #2873; Phospho-ATM (Ser1981) (D25E5) Rabbit mAb #13050; AMPKα #2532; Phospho-AMPKα (Thr172) (D4D6D) Rabbit mAb #50081; mTOR #2972; Phospho-mTOR (Ser2448) #2971; Phospho-mTOR (Ser2481) #2974; ULK1 (R600) #4773; Phospho-ULK1 (Ser317) #37762; Phospho-ULK1 (Ser757) #6888; eIF2α #9722; Phospho-eIF2α (Ser51) #9721; PERK (D11A8) Rabbit mAb #5683; Phospho-PERK (Thr980) (16F8) Rabbit mAb #3179; AKT Antibody #9172; Phospho-AKT (Thr308) (244F9) Rabbit mAb #4056; STAT3 (124H6) Mouse mAb #9139; Phospho-STAT3 (Tyr705) Antibody #9131; STAT5 (D2O6Y) Rabbit mAb #94205; Phospho-STAT5 (Tyr694) #9351; Beclin-1 #3738; ATG5 (D5F5U) Rabbit mAb #12994; ATG13 (D4P1K) Rabbit mAb #13273; Phospho-ATG13 (Ser355) (E4D3T) Rabbit mAb #46329; GRP78/BiP #3183; CHOP (L63F7) Mouse mAb #2895 PP1α Antibody #2582; NFκB p65 (L8F6) Mouse mAb #6956; Phospho-NFκB p65 (Ser536) (93H1) Rabbit mAb #3033; Src (36D10) Rabbit mAb #2109; Phospho-Src Family (Tyr416) (E6G4R) Rabbit mAb #59548; Phospho-Src (Tyr527) Antibody #2015; c-MET (25H2) Mouse mAb # 3127; Phospho-MET (Tyr1234/1235) Antibody #3126; FAS (4C3) Mouse mAb #8023; FAS-L (D1N5E) Rabbit mAb #68405; JAK1/2 (6G4) Rabbit mAb #3344; Phospho-Jak1 (Tyr1034/1035)/Jak2 (Tyr1007/1008) (E9Y7V) Mouse mAb #66245; c-KIT (D13A2) XP^®^ Rabbit mAb #3074; Phospho-c-KIT (Tyr719) Antibody #3391; HER/ErbB Family Antibody Sampler Kit #8339; p70 S6 Kinase #9202; Phospho-p70 S6 Kinase (Thr389) #2904; PDGF Receptor beta #3164; Phospho-PDGF Receptor beta (Tyr754) (23B2) Rabbit mAb #2992; Phospho-p44/42 MAPK (Erk1/2) (Thr202/Tyr204) (20G11) Rabbit mAb #4376; Histone Deacetylase (HDAC) Antibody Sampler Kit #9928; HDAC7 (D4E1L) Rabbit mAb #33418; HDAC8 (E7F5K) Rabbit mAb #66042; HDAC11 (D5I8E) Rabbit mAb #58442; MHC Class II (LGII-612.14) Mouse mAb #68258; p38 MAPK #9212; Phospho-p38 MAPK (Thr180/Tyr182) (3D7) Rabbit mAb #9215; LATS1 (C66B5) Rabbit mAb #3477; Phospho-LATS1/2 (Ser909) #9157; Phospho-LATS1/2 (Thr1079) (D57D3) Rabbit mAb #8654; YAP (1A12) Mouse mAb #12395; Phospho-YAP (Ser127) (D9W2I) Rabbit mAb #13008; Phospho-YAP (Ser109) (E5I9G) Rabbit mAb #53749; Phospho-YAP (Ser397) (D1E7Y) Rabbit mAb #13619; TAZ (E8E9G) Rabbit mAb #83669 Phospho-TAZ (Ser89) (E1X9C) Rabbit mAb #59971; NEDD4 Antibody #2740; PTEN Antibody #9552; Estrogen Receptor α (D6R2W) Rabbit mAb #13258; Cyclin Antibody Sampler Kit #9869; BCL-XL #2762; MCL-1 (D35A5) Rabbit mAb #5453; BAX #2772; BAK #2814; BIM #2819; JNK1/2 #9252; Phospho-JNK (Thr183/Tyr185) (81E11) Rabbit mAb #4668; p44/42 MAPK (ERK1/2) (L34F12) Mouse mAb #4696). Santa Cruz Biotechnology antibodies: Histone Deacetylase 9 (HDAC9) (B-1) #sc398003; Histone Deacetylase 10 (HDAC10) (E-2) #393417. ABCAM antibodies: Anti-PD-L1 [28–8] (ab205921); Anti-PD-L2 [EPR25200-50] (ab288298); Anti-Ornithine Decarboxylase/ODC [ODC1/2878R] (ab270268); BAG3 ab92309; HSP90 (#2928); HSP90 (ab195575); HSP90 3G3 (13495); GRP78 (ab191023); GRP78 (ab103336); HSP27 [EP1724Y] (ab62339). Specific multiple independent siRNAs to knock down expression were purchased from Qiagen (Hilden, Germany). Human: HSP90 GeneGlobe ID SI03028606; HSP70 GeneGlobe ID SI04324481; GRP78 GeneGlobe ID SI00443114; Beclin-1 GeneGlobe ID SI00055573; ATG5 GeneGlobe ID SI00069251; Rubicon GeneGlobe ID SI00452592; BAG3 GeneGlobe ID SI02632812; AMPKα1 GeneGlobe ID SI00086387; eIF2α GeneGlobe ID SI00105784; ULK1 GeneGlobe ID SI00053060; perk GeneGlobe ID SI00069048. Mouse: Beclin-1 GeneGlobe ID SI00214165; ATG5 GeneGlobe ID SI00230664; BAG3 GeneGlobe ID SI00208425; AMPKα1 GeneGlobe ID SI01388247; eIF2α GeneGlobe ID SI00969675; ULK1 GeneGlobe ID SI01461999; PERK GeneGlobe ID SI00991319. Thermo Fisher mouse: HSP70 si RNA ID: s201487 Cat #4390771; GRP78 si RNA ID: s67084 Cat #4390771; Rubicon si RNA ID: s104761 Cat #4390771; HSP90 si RNA ID: s67897 Cat #4390771. Control studies were presented showing on-target specificity of our siRNAs, primary antibodies, and our phospho-specific antibodies to detect both total protein levels and phosphorylated levels of proteins (1–6) (**Table 1**). Please see references 1 and 6 to observe representative images of the scanned cells.

**Table 1:** Control data showing siRNA knock down and protein over-expression.

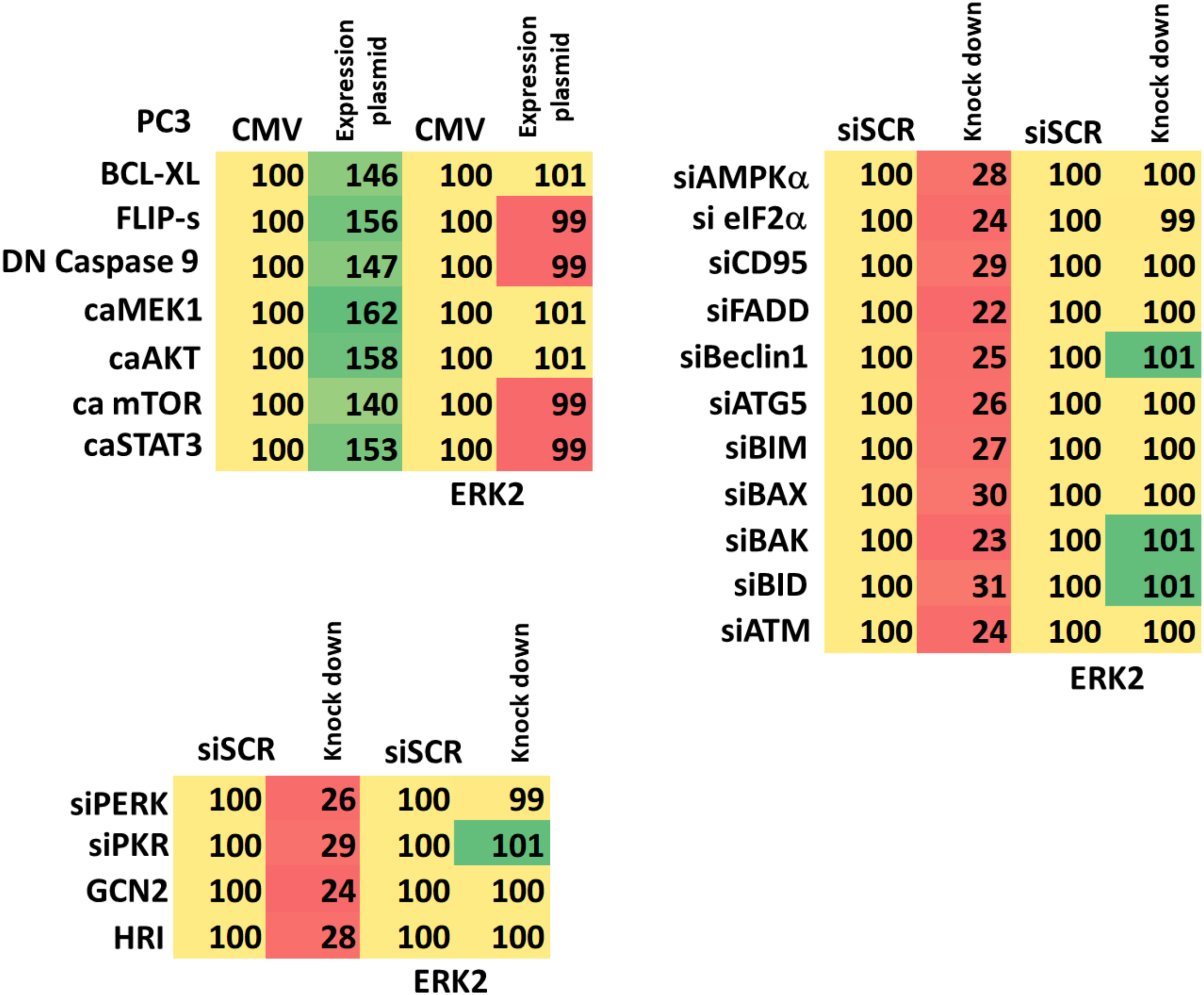

PC3 cells as indicated were transfected with siRNA molecules to knock down the expression of the indicated proteins or transfected with plasmids to over-express the indicated proteins. The percentage remaining after knock-down or the percentage over-expression above basal levels is indicated. (n = 3 +/-SD) (total ERK2 is included as an invariant total protein loading control).

### Methods

All bench-side Methods used in this manuscript have been previously performed and described in the peer-reviewed references (1–6). Briefly, cells were grown at 37°C (5% (v/v CO_2_) using RPMI supplemented 5% (v/v) fetal calf serum and 1% (v/v) Non-essential amino acids. All therapeutics were dissolved in DMSO making a 10 mM stock solution, stored in multiple 100 μl vials. The final concentration of DMSO is never more than 0.1% (v/v). Cells were not cultured in reduced serum media.

### Assessments of protein expression and protein phosphorylation

Multi-channel fluorescence HCS microscopes perform true in-cell western blotting. Three independent cultures derived from three thawed vials of cells of a tumor were sub-cultured into individual 96-well plates (~5,000 cells per well). Twenty-four hours after plating, the cells are transfected with a control plasmid or a control siRNA, or with an empty vector plasmid or with plasmids to express various proteins. After another 24 hours, the cells are ready for drug exposure(s). At various time-points after the initiation of drug exposure, cells are fixed in place using paraformaldehyde and using Triton X100 for permeabilization. Primary antibodies (1:500 dilution for all experiments) are used, and plates are incubated at 4°C overnight with gentle rocking. Cells are washed three times with PBS and then subsequently incubated with validated fluorescent-tagged secondary antibodies (1:1000) for 30 min. Cells are then washed an additional three times with PBS. The microscope determines the background fluorescence in the well and in parallel randomly determines the mean fluorescent intensity of 100 cells per well. The counting is independent of cell density. Of note for scientific rigor is that the operator does not personally manipulate the microscope to examine specific cells; the entire fluorescent accrual method is independent of the operator. For representative images of cells that are typical of this process, please see references 1 and 6 (1–6).

### Detection of cell death by trypan blue assay

Cells were treated with vehicle control or with drugs alone or in combination for 24h. At the indicated time points cells were harvested by trypsinization and centrifugation. Cell pellets were resuspended in PBS and mixed with trypan blue agent. Viability was determined microscopically using a hemocytometer. Five hundred cells from randomly chosen fields were counted and the number of dead cells was counted and expressed as a percentage of the total number of cells counted (1–6).

### Transfection of cells with siRNA or with plasmids

Cells were plated and 24h after plating, transfected. Plasmids to express FLIP-s, BCL-XL, dominant negative caspase 9, activated AKT, activated STAT3, activated mTOR and activated MEK1 EE were used throughout the study (Addgene, Waltham, MA). Empty vector plasmid (CMV) was used as a control. For siRNA transfection, 10 nM of the annealed siRNA or the negative control (a “scrambled” sequence with no significant homology to any known gene sequences from mouse, rat or human cell lines) were used (1–6).

### Assessments of autophagosome and autolysosome levels

Cells were transfected with a plasmid to express LC3-GFP-RFP (Addgene, Watertown MA). Twenty-four hs after transfection, cells are treated with vehicle control or the drugs alone or in combination. Cells were imaged and recorded at 60X magnification 4h and 8h after drug exposure and the mean number of [GFP+RFP+] and [RFP+] punctae per cell determined from >50 randomly selected cells per condition (1–6).

### Comet assays

Drug-treated cells, in suspension, are mixed with low melting point agarose and spread onto a microscope glass slide. After lysis of cells with detergent at a high salt concentration, DNA unwinding and electrophoresis was carried out at neutral pH (7-8). Unwinding of the DNA and electrophoresis at neutral pH permits the detection of double strand breaks and cross links. Collins et al. published a visual scoring method that classifies comets from grades 0–4 (11–13). One hundred comets were scored, and each comet assigned a value of 0 to 4 according to its class, the mean total score for the sample gel will be between 0 and 4 “arbitrary units.”

### Animal studies

Studies were performed according to the U.S. Department of Agriculture regulations under the VCU IACUC protocol AD20008. Male NRG mice supplied by the Massey Cancer Center Animal Core (∼20 g) were injected with 1.0×10^6^ male LNCaP cells into their rear flank (10 animals per treatment group). Tumors were permitted to form for 1 week with tumors at that time exhibiting a mean volume of approximately 50 mm^3^. Mice were treated by oral gavage once every day for 45 days with vehicle control, GZ17‐6.02 (50 mg/kg), olaparib (10 mg/kg) or the drugs in combination. Before, during and after drug treatment tumors were calipered every ~3 days as indicated in the Figure and tumor volume was assessed up to 45 days later. Tumor volumes under each condition are plotted. Animals were humanely killed when the tumor volume reached approximately 2,000 mm^3^ due to ulceration. Animal survival was plotted on a Kaplan–Meier curve. Animal body mass under all treatment conditions did not significantly change over the 45-day time course.

### Data analysis

Comparison of the effects of various treatments was using one-way ANOVA for normalcy followed by a two tailed Student’s t-test with multiple comparisons. *In vivo* animal survival data utilizes log rank statistical analyses for comparison between the survival of different treatment groups. Differences with a p-value of < 0.05 were considered statistically significant. Experiments are the means of multiple individual data points per experiment from 3 independent experiments (± SD).

## Results

**Figure 1:**
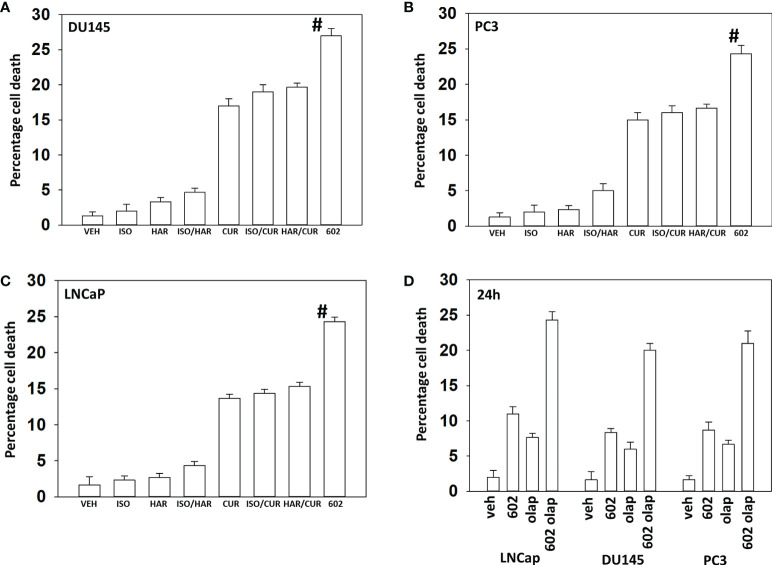
GZ17-6.02 interacts with olaparib to kill prostate cancer cells. **(A–C)** Prostate cancer cells were treated with vehicle control, GZ17-6.02 [curcumin (2.0 μM) + harmine (4.5 μM) + isovanillin (37.2 μM)] or with component parts of GZ17-6.02 as individual agents at the indicated concentrations or in duo combinations. Cells were isolated 48h afterwards and viability determined *via* trypan blue exclusion assays (n = 3 +/- SD). # p < 0.05 greater than other tested drug treatments. **(D)** DU145, PC3 and LNCaP prostate cancer cells were treated with vehicle control, GZ17-6.02 (2 μM), olaparib (50 nM) or the drugs in combination for 24h. Cells were isolated, and viability determined by trypan blue exclusion. (n = 3 +/- SD).

Compared to its individual component parts, as single agents or together in pairs, GZ17-6.02 was the most efficacious agent at killing prostate cancer cells (**Figures 1A–C**). We then performed studies to define the biology of GZ17-6.02 and the PARP1 inhibitor olaparib alone and in combination in prostate cancer cells. GZ17-6.02 and olaparib interacted in an arithmetically greater than additive fashion with olaparib to kill prostate cancer cells (**Figure 1D**).

We then examined the impact of GZ17-6.02 and olaparib on cell signaling and protein expression levels in the three cell lines (**Tables 2**-**4**). Compared to prior studies in other tumor cell types such as pancreatic, liver, or colorectal, we observed significantly greater activation of ATM and the AMPK (p < 0.05). The phosphorylation of eIF2α was also robustly enhanced in the prostate cancer cell lines compared to GI tumor types, yet in contrast to prior work in other tumor cell types, the phosphorylation of PKR-like endoplasmic reticulum kinase (PERK) was not significantly elevated. Other indicators of a strong ER stress response, including elevated expression of Beclin1, ATG5, and GRP78 were evident, whereas the levels of CHOP were only enhanced in PC3 cells. In all three lines, the drug combination reduced expression of HDAC2, HDAC3 and HDAC6. LNCaP and PC3 cells do not express PTEN, yet the drug combination inactivated AKT, mTOR and p70 S6K in these lines as well as in DU145 cells. Unlike other tumor cell types previously tested, in all three prostate lines, the drug combination significantly enhanced activity in the c-Jun NH2-terminal kinase (JNK) pathway.

**Table 2:** Regulation of cell signaling by GZ17-6.02 and olaparib in LNCaP prostate cancer cells.

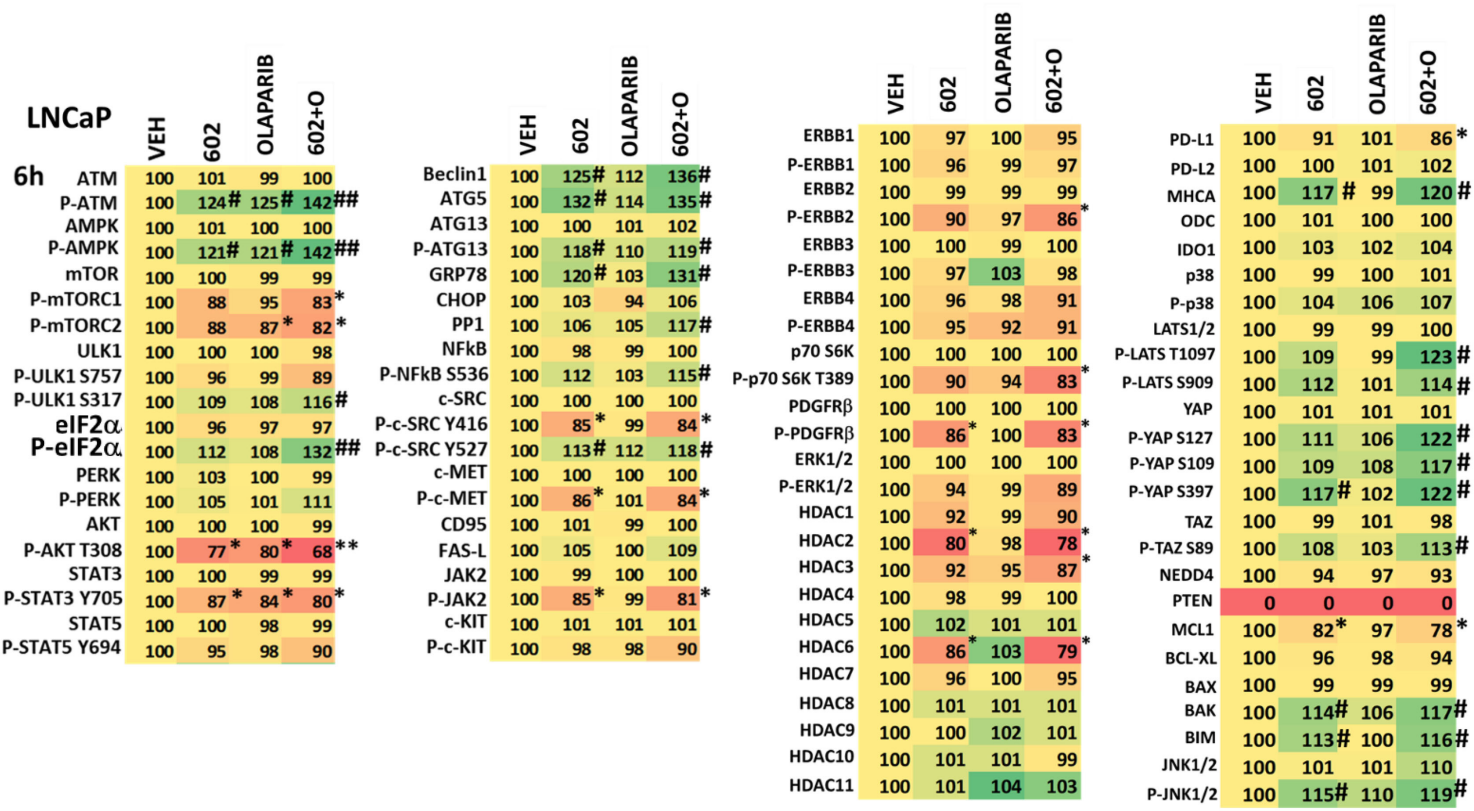

LNCaP cells were treated with vehicle control, GZ17-6.02 (2 μM), olaparib (50 nM) or the drugs in combination for 6h. Cells were fixed in situ, permeabilized, stained with the indicated validated primary antibodies and imaged with secondary antibodies carrying red- and green-fluorescent tags. The staining intensity of at least 100 cells per well/condition is determine in three separate studies. The data are the normalized amount of fluorescence set at 100% comparing intensity values for vehicle control (n = 3 +/-SD). # p < 0.05 greater than vehicle control; * p < 0.05 less than vehicle; ## = greater than GZ17-6.02 as a single agent; ** = less than GZ17-6.02 as a single agent.

**Table 3:** Regulation of cell signaling by GZ17-6.02 and olaparib in PC3 prostate cancer cells.

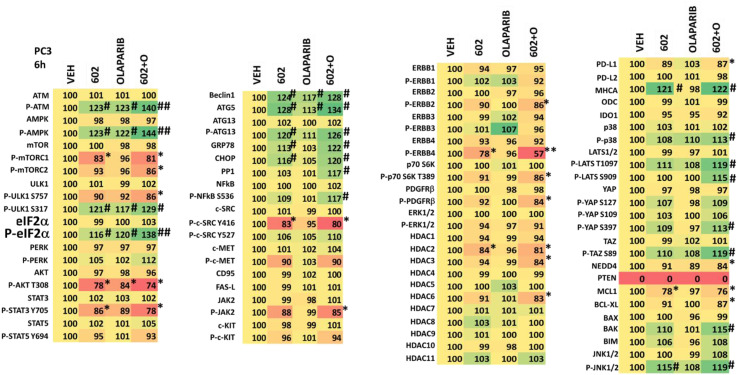

PC3 cells were treated with vehicle control, GZ17-6.02 (2 μM), olaparib (50 nM) or the drugs in combination for 6h. Cells were fixed in situ, permeabilized, stained with the indicated validated primary antibodies and imaged with secondary antibodies carrying red- and green-fluorescent tags. The staining intensity of at least 100 cells per well/condition is determine in three separate studies. The data are the normalized amount of fluorescence set at 100% comparing intensity values for vehicle control (n = 3 +/-SD). # p < 0.05 greater than vehicle control; * p < 0.05 less than vehicle. ## = greater than GZ17-6.02 as a single agent; ** = less than GZ17-6.02 as a single agent.

**Table 4:** Regulation of cell signaling by GZ17-6.02 and olaparib in DU145 prostate cancer cells.

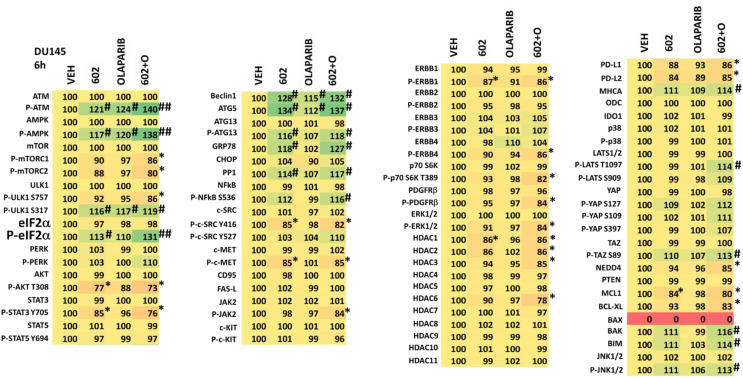

DU145 cells were treated with vehicle control, GZ17-6.02 (2 μM), olaparib (50 nM) or the drugs in combination for 6h. Cells were fixed in situ, permeabilized, stained with the indicated validated primary antibodies and imaged with secondary antibodies carrying red- and green-fluorescent tags. The staining intensity of at least 100 cells per well/condition is determine in three separate studies. The data are the normalized amount of fluorescence set at 100% comparing intensity values for vehicle control (n = 3 +/-SD). # p < 0.05 greater than vehicle control; * p < 0.05 less than vehicle. ## = greater than GZ17-6.02 as a single agent; ** = less than GZ17-6.02 as a single agent.

**Figure 2:**
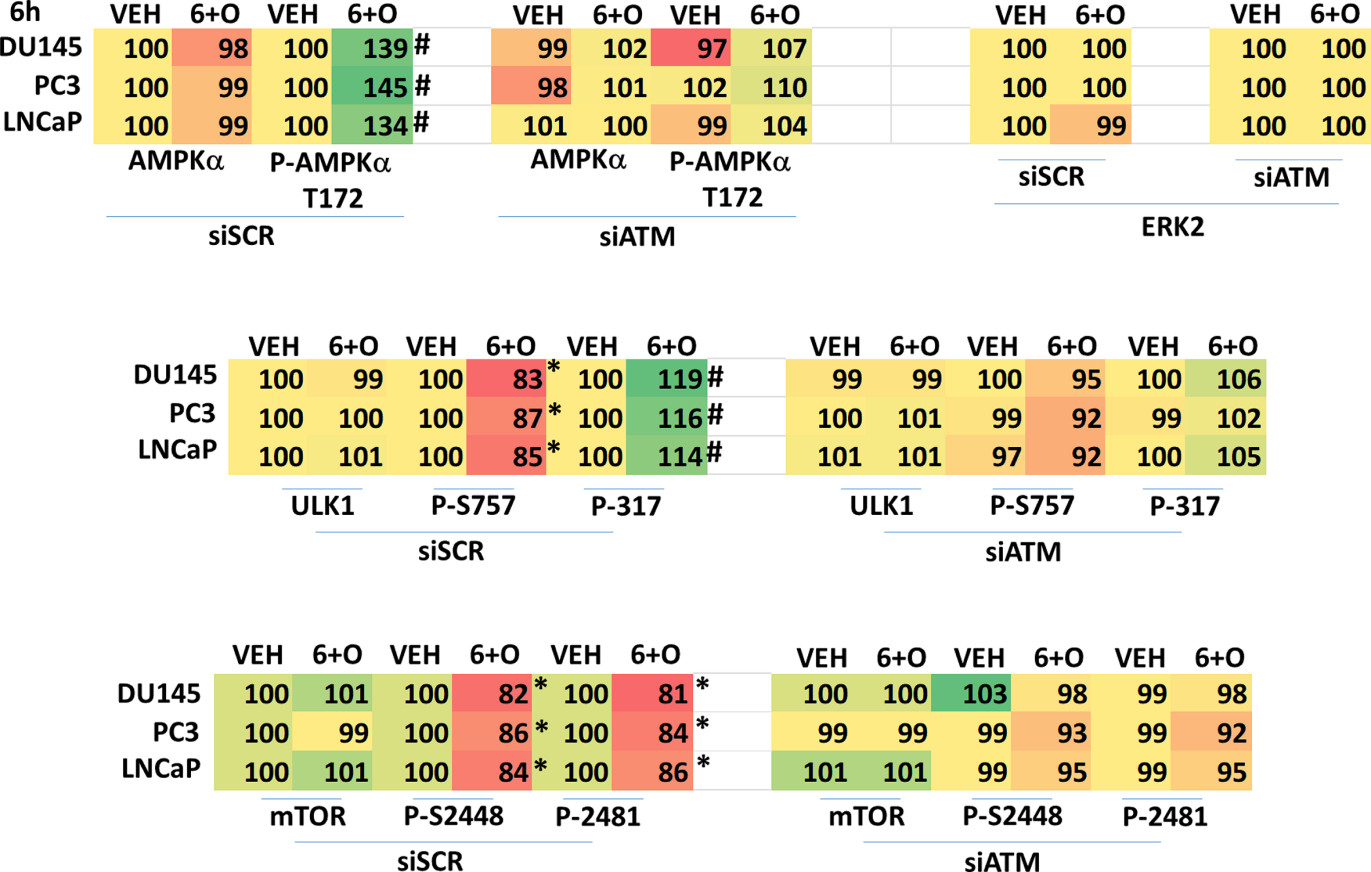
Regulation of AMPK, ULK1 and mTOR by [GZ17-6.02 + olaparib] requires ATM. Cells were transfected with a scrambled siRNA or with an siRNA to knock down expression of ATM. After 24h, cells were treated with vehicle control or [GZ17-6.02 (2 μM) and olaparib (50 nM)] in combination for 6h. Cells were fixed in situ, permeabilized, stained with the indicated validated primary antibodies and imaged with secondary antibodies carrying red- and green-fluorescent tags. The staining intensity of at least 100 cells per well/condition is determined in three separate studies. The graphical data presented are the normalized amount of fluorescence set at 100% comparing intensity values for vehicle control (n = 3 +/-SD). # p < 0.05 greater than vehicle control; * p < 0.05 less than vehicle control.

**Figure 3:**
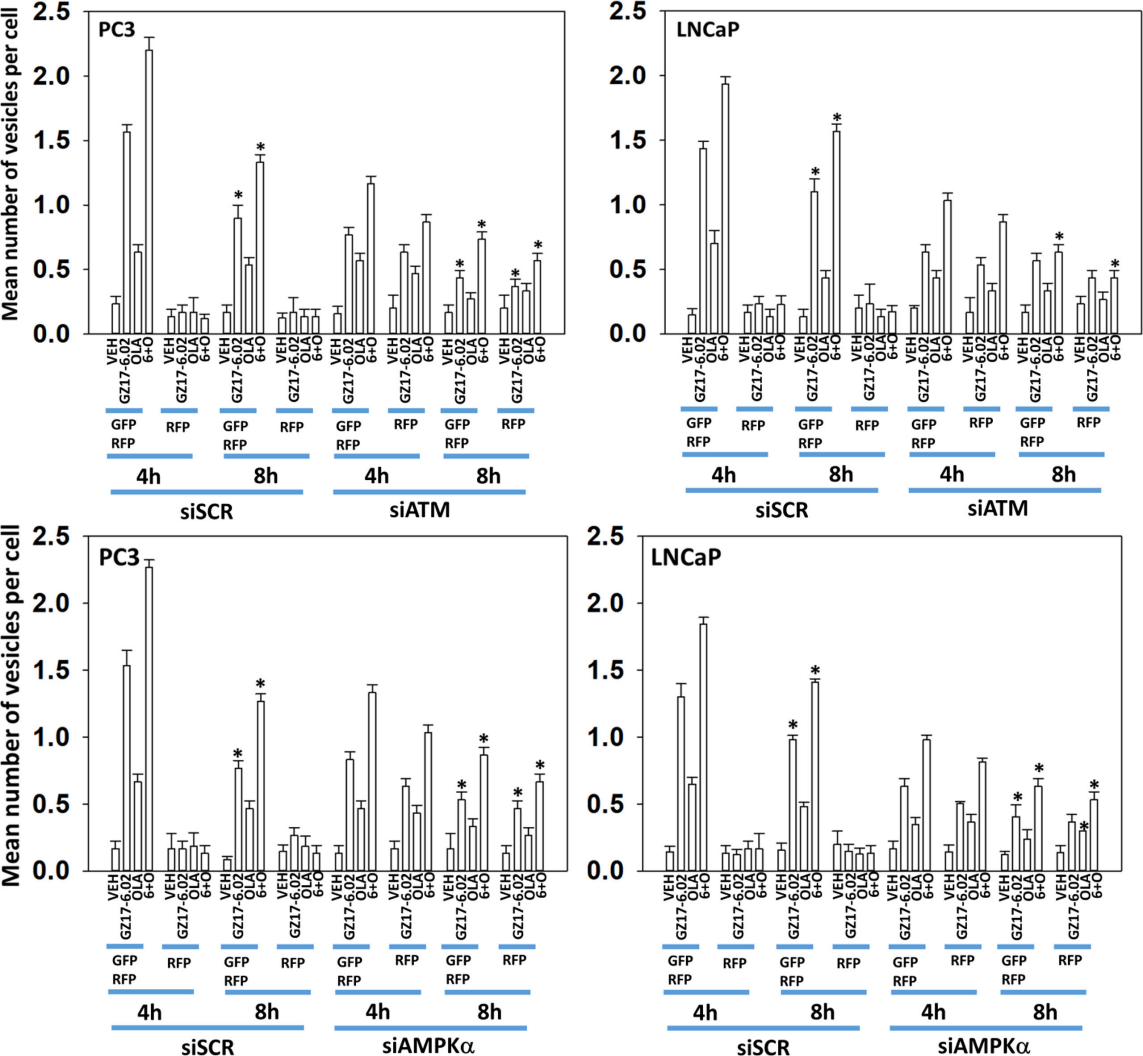
Knock down of ATM reduces autophagosome formation and autophagic flux. PC3 and LNCaP cells were transfected with a plasmid to express LC3-GFP-RFP and in parallel transfected with a scrambled siRNA or with siRNA molecules to knock down the expression of ATM or of AMPKα. Twenty-four h later, cells were treated with vehicle control, GZ17-6.02 (2 μM), olaparib (50 nM) or the drugs in combination for 4h and 8h. At each time point, the mean number of GFP+RFP+ and only RFP+ vesicles per cell were determined (n = 3 +/-SD). * p < 0.05 less than corresponding value in siSCR transfected cells.

**Figure 4:**
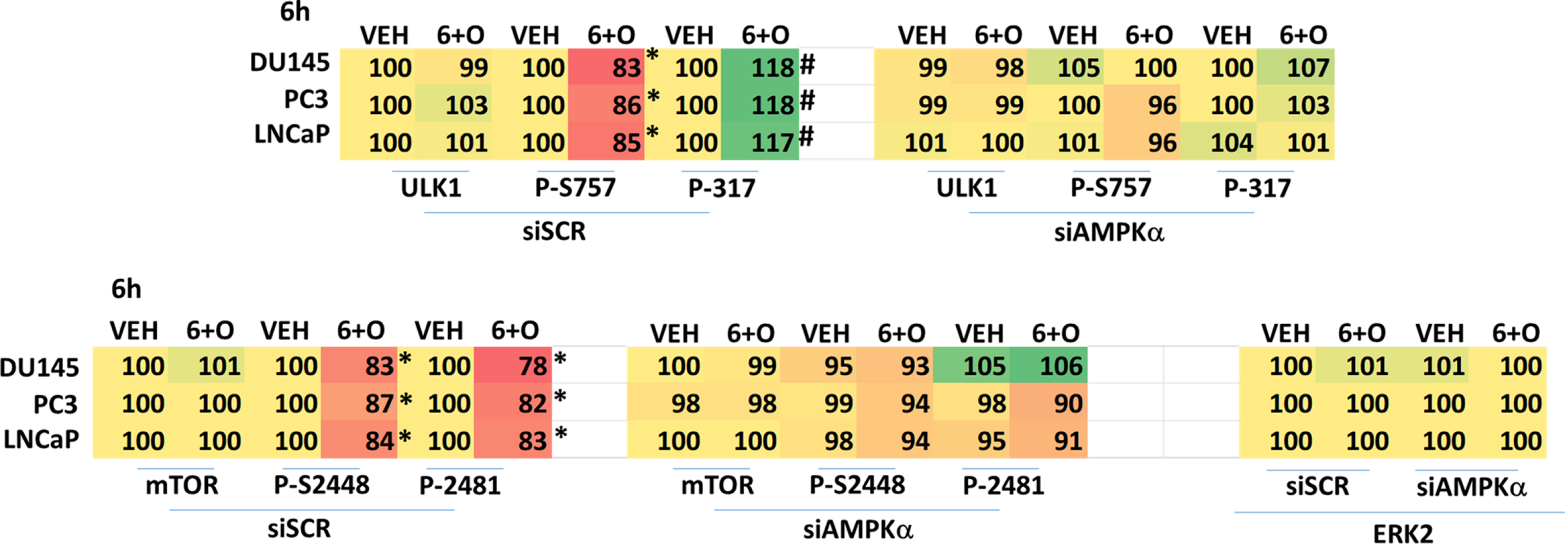
Regulation of ULK1 and mTOR by [GZ17-6.02 + olaparib] requires the AMPK. Cells were transfected with a scrambled siRNA or with an siRNA to knock down expression of AMPKα. After 24h, cells were treated with vehicle control or [GZ17-6.02 (2 μM) and olaparib (50 nM)] in combination for 6h. Cells were fixed in situ, permeabilized, stained with the indicated validated primary antibodies and imaged with secondary antibodies carrying red- and green-fluorescent tags. The staining intensity of at least 100 cells per well/condition is determined in three separate studies. The data are the normalized amount of fluorescence set at 100% comparing intensity values for vehicle control (n = 3 +/-SD). # p < 0.05 greater than vehicle control; *p < 0.05 less than vehicle control.

We next performed molecular studies to link the cause-and-effect of cell signaling processes outlined in **Figures 2**–**4**. Knock down of ATM significantly reduced the ability of the drug combination to increase phosphorylation of AMPKα T172 and ULK1 S317, and to decrease the phosphorylation of ULK1 S757, mTOR S2448 and mTOR S2481 (**Figure 2**). In agreement with these changes in protein phosphorylation, knock down of ATM or of the AMPKα significantly reduced autophagosome formation and reduced levels of autophagic flux (**Figure 3**). Knock down of AMPKα prevented the drug combination from altering the phosphorylation of ULK1 or mTOR (**Figure 4**). Thus, GZ17-6.02 -induced ATM-AMPK signaling regulates the activities of ULK1 and mTOR.

**Figure 5:**
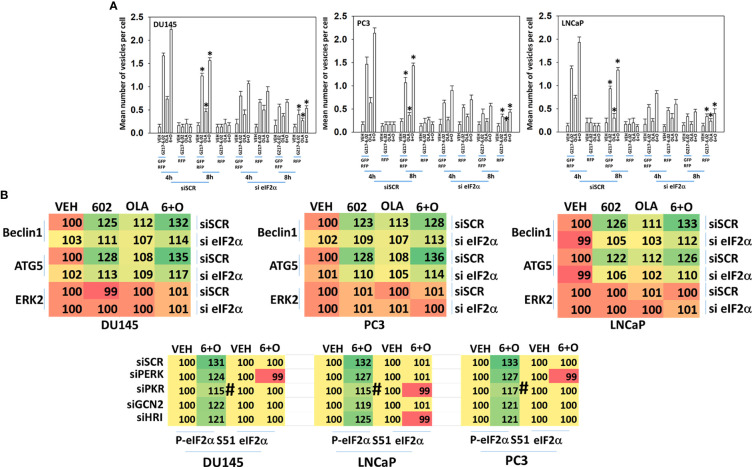
ER stress signaling is required for autophagosome formation and the increased expression of Beclin1 and ATG5. **(A)** Cells were transfected with a plasmid to express LC3-GFP-RFP and in parallel transfected with a scrambled siRNA or with an siRNA to knock down the expression of eIF2α. Twenty-four h later, cells were treated with vehicle control, GZ17-6.02 (2 μM), olaparib (50 nM) or the drugs in combination for 4h and 8h. At each time point, the mean number of GFP+RFP+ and only RFP+ vesicles per cell were determined (n = 3 +/-SD). *p < 0.05 less than corresponding value in siSCR transfected cells. **(B)** Cells were transfected with a scrambled siRNA or with an siRNA to knock down the expression of eIF2α. Twenty-four h later, cells were treated with vehicle control, GZ17-6.02 (2 μM), olaparib (50 nM) or the drugs in combination for 6h. Cells were fixed in situ, permeabilized, stained with the indicated validated primary antibodies and imaged with secondary antibodies carrying red- and green-fluorescent tags. The staining intensity of at least 100 cells per well/condition is determined in three separate studies. The data presented the normalized amount of fluorescence set at 100% comparing intensity values for vehicle control (n = 3 +/-SD). # p < 0.05 greater than vehicle control; *p < 0.05 less than vehicle control; † p < 0.05 less than corresponding value in siSCR cells.

Knock down of eIF2α reduced the abilities of GZ17-6.02 and olaparib to increase autophagosome formation and to flux into autolysosomes and knock down of eIF2α significantly reduced the ability of GZ17-6.02 alone or in combination with olaparib to increase Beclin1 and ATG5 expression, proteins that are essential for autophagosome formation (**Figures 5A, B**). In **Table 2**, although drug exposure significantly increased eIF2α S51 phosphorylation, PERK was not significantly activated and hence we determined the most likely kinase(s) regulating eIF2α S51 phosphorylation in prostate cancer cells. There are several other well-described kinases that catalyze the phosphorylation of eIF2α S51, including the interferon-induced, double-stranded RNA-activated protein kinase, known as protein kinase R (PKR); general control non-derepessible 2 (GCN2); and heme-regulated eIF2α kinase (HRI) (14, 15). Across all three cell lines tested, only knock down of PKR significantly reduced the drug-induced enhancement of eIF2α S51 phosphorylation (**Figure 5C**).

**Figure 6:**
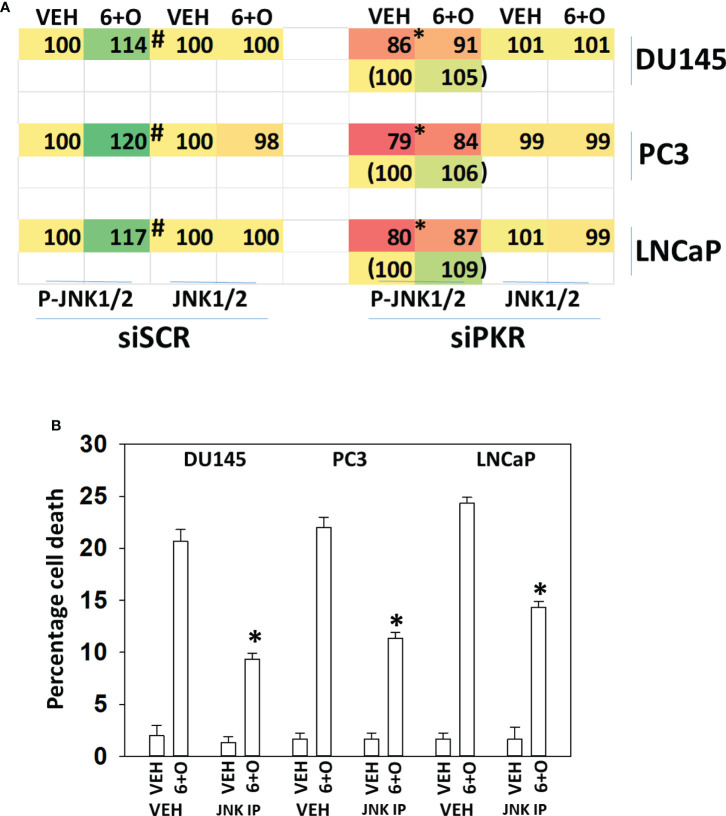
Activation of toxic JNK1/2 signaling by GZ17-6.02 and olaparib requires PKR. **(A)** Prostate cancer cells were transfected with a scrambled siRNA molecule or with an siRNA to knock down PKR expression. Twenty-four h later, cells were treated with vehicle control or with [GZ17-6.02 (2 μM) + olaparib (50 nM)] for 6h. Cells were fixed in place and the phosphorylation and total expression of JNK1/2 determined. (n = 3 +/-SD) #p < 0.05 greater than corresponding vehicle control; *less than corresponding value in siSCR cells. **(B)** Prostate cancer cells were treated with vehicle control or with [GZ17-6.02 (2 μM) + olaparib (50 nM)] for 24h in the presence of vehicle (DMSO) or the molecular cell-permeant JNK inhibitory peptide (10 μM). Cells were isolated and viability determined by trypan blue exclusion assay (n = 3 +/-SD) * p < 0.05 less than corresponding value in vehicle control treated cells.

**Figure 7:**
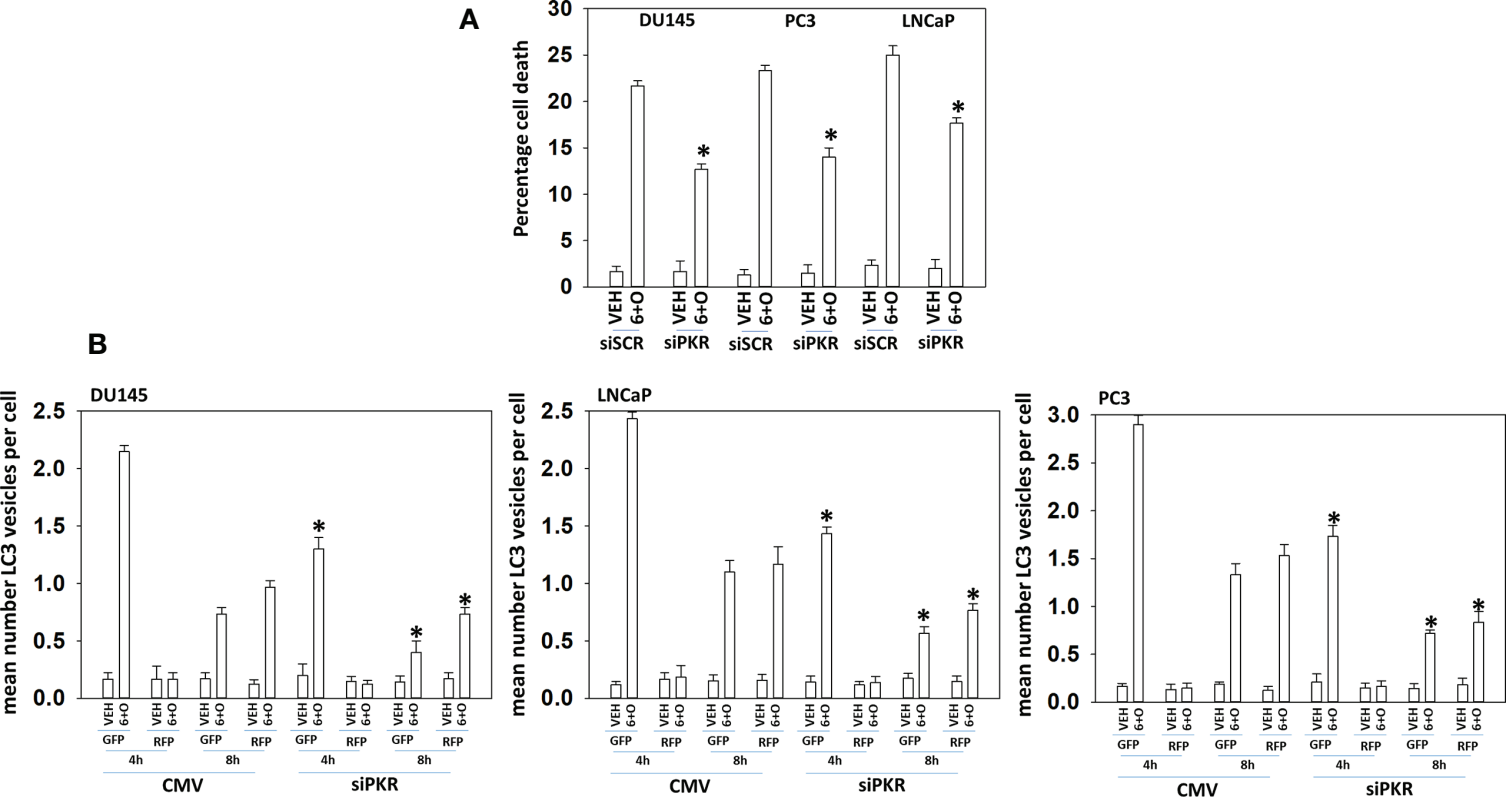
Signaling by PKR is required for tumor cell killing and autophagosome formation. **(A)** Prostate cancer cells were transfected with a scrambled siRNA or with an siRNA to knock down the expression of PKR. Twenty-four h later, cells were treated with vehicle control or with [GZ17-6.02 (2 μM) + olaparib (50 nM)] for 24h. Cells were isolated, and viability determined by trypan blue exclusion assay (n = 3 +/-SD) *p < 0.05 less than corresponding value in vehicle control treated cells. **(B)** Prostate cancer cells were transfected with a scrambled siRNA or with an siRNA to knock down the expression of PKR and in parallel transfected with a plasmid to express LC3-GFP-RFP. Twenty-four h later, cells were treated with vehicle control, GZ17-6.02 (2 μM), olaparib (50 nM) or the drugs in combination for 4h and 8h. At each time point, the mean number of GFP+RFP+ and only RFP+ vesicles per cell were determined (n = 3 +/-SD). *p < 0.05 less than corresponding value in siSCR transfected cells.

Signaling by PKR can activate the JNK1/2 MAPK pathway. As eIF2α S51 phosphorylation was predominantly mediated by PKR in prostate cancer cells, and that we had unexpectedly observed activation of the JNK1/2 pathway in prostate cancer cells, we determined whether the JNK1/2 pathway was being regulated by GZ17-6.02 *via* a PKR-dependent mechanism. Knock down of PKR reduced the basal activity of the JNK1/2 pathway by ~20% and knock down of PKR largely prevented the drug combination from activating JNK1/2 (**Figure 6A**). Treatment of cells with the cell-permeable JNK-inhibitory peptide significantly reduced the lethality of the drug combination (**Figure 6B**). Knock down of PKR significantly reduced the lethality of the drug combination (**Figure 7A**). Knock down of PKR also reduced the amount of drug-induced autophagosome formation but did not block autophagic flux (**Figure 7B**).

**Figure 8:**
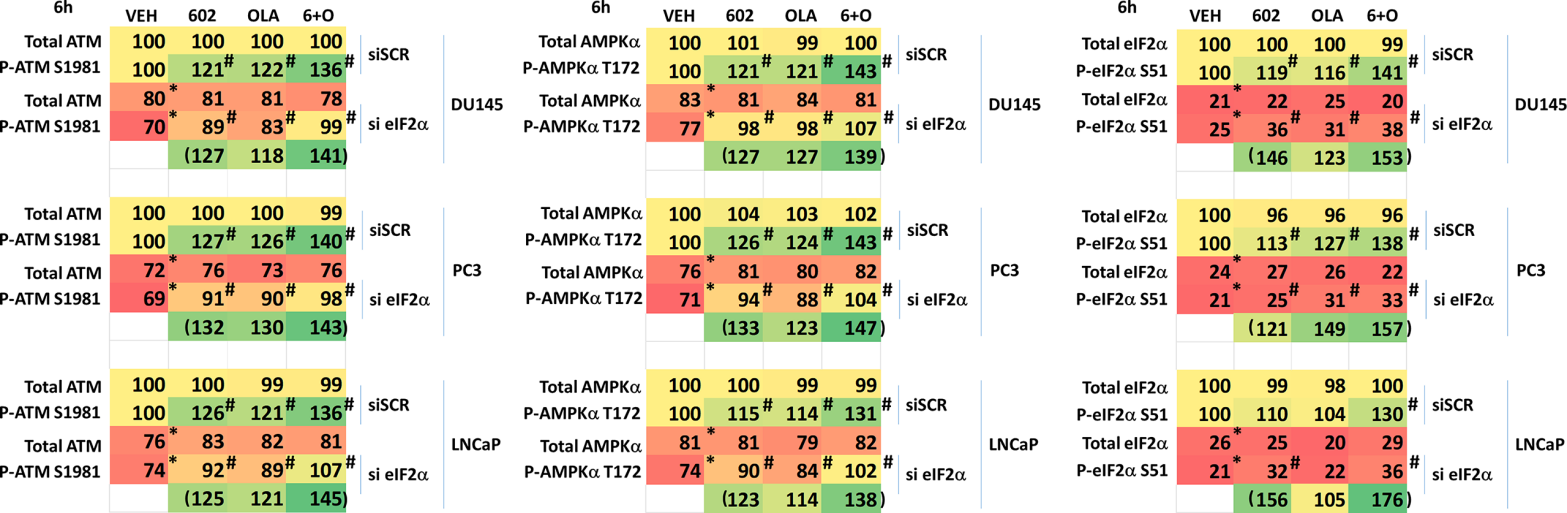
Activation of ATM and the AMPK by GZ17-6.02 alone or in combination with olaparib requires ER stress signaling. Cells were transfected with a scrambled siRNA or with an siRNA to knock down the expression of eIF2α. After 24h, cells were treated with vehicle control, GZ17-6.02 (2 μM), olaparib (50 nM) or the drugs in combination for 6h. Cells were fixed in situ, permeabilized, stained with the indicated validated primary antibodies and imaged with secondary antibodies carrying red- and green-fluorescent tags. The staining intensity of at least 100 cells per well/condition is determined in three separate studies. The data are the normalized amount of fluorescence set at 100% comparing intensity values for vehicle control (n = 3 +/-SD). #p < 0.05 greater than vehicle control; *p < 0.05 less than vehicle control.

**Figure 9:**
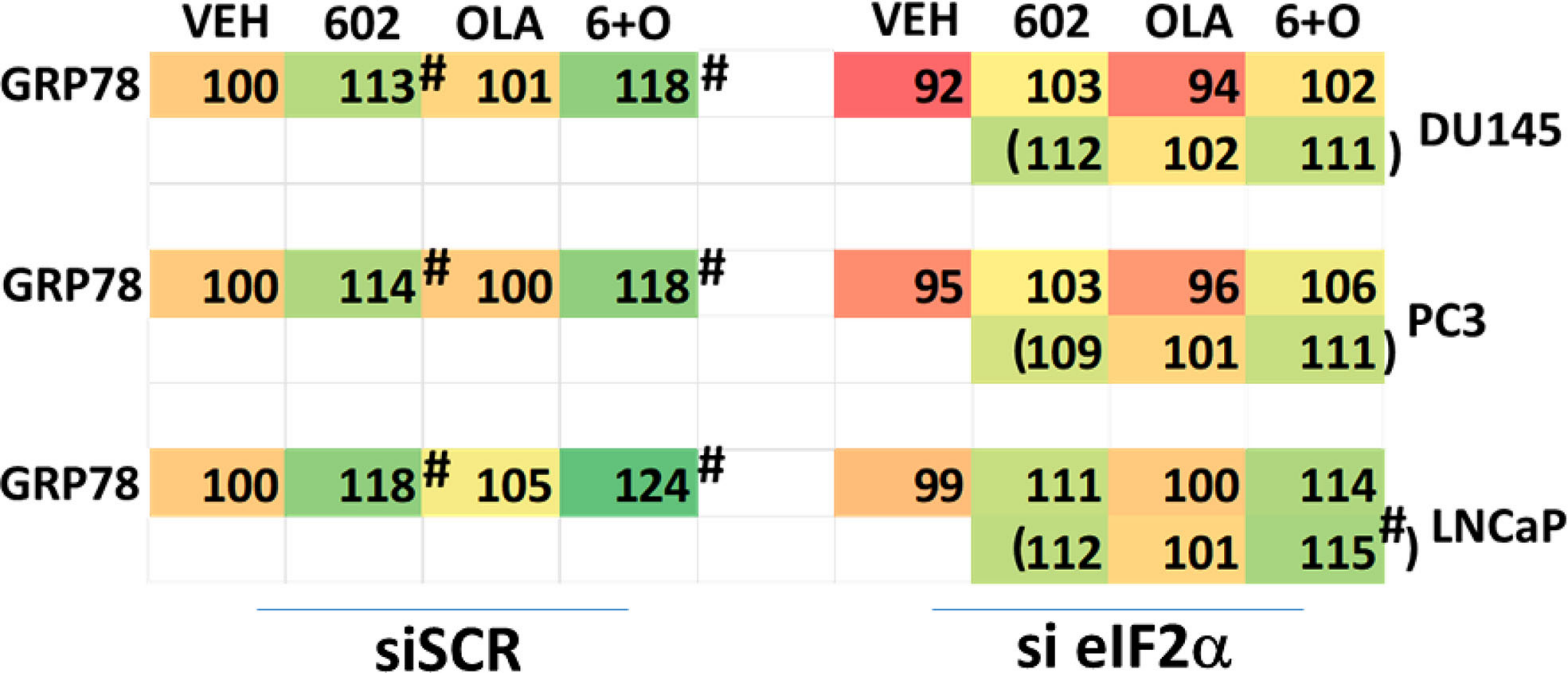
Increased expression of GRP78 caused by GZ17-6.02 alone or in combination with olaparib requires ER stress signaling. Cells were transfected with a scrambled siRNA or with an siRNA to knock down the expression of eIF2α. After 24h, cells were treated with vehicle control, GZ17-6.02 (2 μM), olaparib (50 nM) or the drugs in combination for 6h. Cells were fixed in situ, permeabilized, stained with the indicated validated primary antibodies and imaged with secondary antibodies carrying red- and green-fluorescent tags. The staining intensity of at least 100 cells per well/condition is determined in three separate studies. The data are the normalized amount of fluorescence set at 100% comparing intensity values for vehicle control (n = 3 +/-SD). #p < 0.05 greater than vehicle control; *p < 0.05 less than vehicle control.

We then defined the impact of knocking down eIF2α expression on the abilities of GZ17-6.02 and olaparib to activate ATM and the AMPK. Knock down of eIF2α unexpectedly reduced the basal protein expression levels of both ATM and AMPKα (**Figure 8**). However, knock down of eIF2α did not alter the *relative ability* of the drugs to cause ATM and AMPK activation or inactivation of residual eIF2α itself. Knock down of eIF2α reduced the ability of GZ17-6.02 as a single agent and when combined with olaparib to significantly increase GRP78 levels, although a trend was evident arguing loss of eIF2α did not fully block the induction of GRP78 (**Figure 9**). The enhanced GRP78 expression was localized in punctate bodies in the cytoplasm of the tumor cells (not shown).

**Figure 10:**
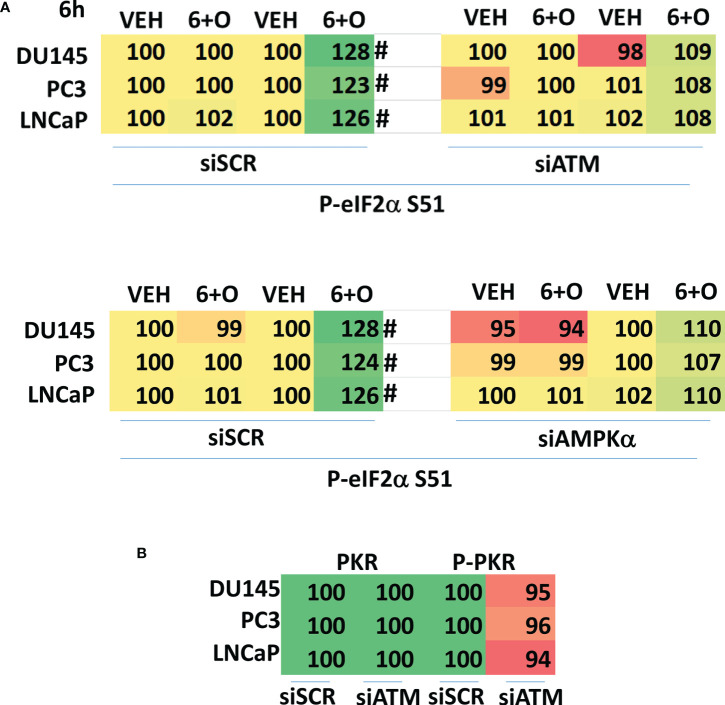
Knock down of ATM or AMPKα reduces the ability of [GZ17-6.02 + olaparib] to increase eIF2α S51 phosphorylation. **(A)** Cells were transfected with a scrambled siRNA control or with siRNA molecules to knock down the expression of either ATM or AMPKα. After 24h, cells were treated with vehicle control or with [GZ17-6.02 (2 μM) and olaparib (50 nM)] in combination for 6h. Cells were fixed in situ, permeabilized, stained with the indicated validated primary antibodies and imaged with secondary antibodies carrying red- and green-fluorescent tags. The staining intensity of at least 100 cells per well/condition is determined in three separate studies. The data are the normalized amount of fluorescence set at 100% comparing intensity values for vehicle control (n = 3 +/-SD). #p < 0.05 greater than vehicle control; *p < 0.05 less than vehicle control. **(B)** Cells were transfected with a scrambled siRNA control or with siRNA molecules to knock down the expression of ATM or AMPKα. After 24h, cells were fixed in place and staining for PKR and ERK2 as a loading control performed (n = 3 +/-SD) * p < 0.05 less than siSCR control.

We then determined whether knock down of ATM or AMPKα altered the drug-induced increase in eIF2α S51 phosphorylation. Knock down of either ATM or AMPKα significantly reduced the ability of the drug combination to enhance phosphorylation of eIF2α S51 (**Figure 10A**). Based on our findings in **Figures 4**-**6A**, we determined whether knock down of ATM altered the basal expression of PKR. Knock down of ATM neither altered PKR expression nor its phosphorylation (**Figure 10B**). Thus, in prostate cancer cells eIF2α controls basal ATM and AMPKα protein levels and that in the absence of ATM or AMPK, the ability of the cell to cause eIF2α S51 phosphorylation is also diminished.

**Figure 11:**
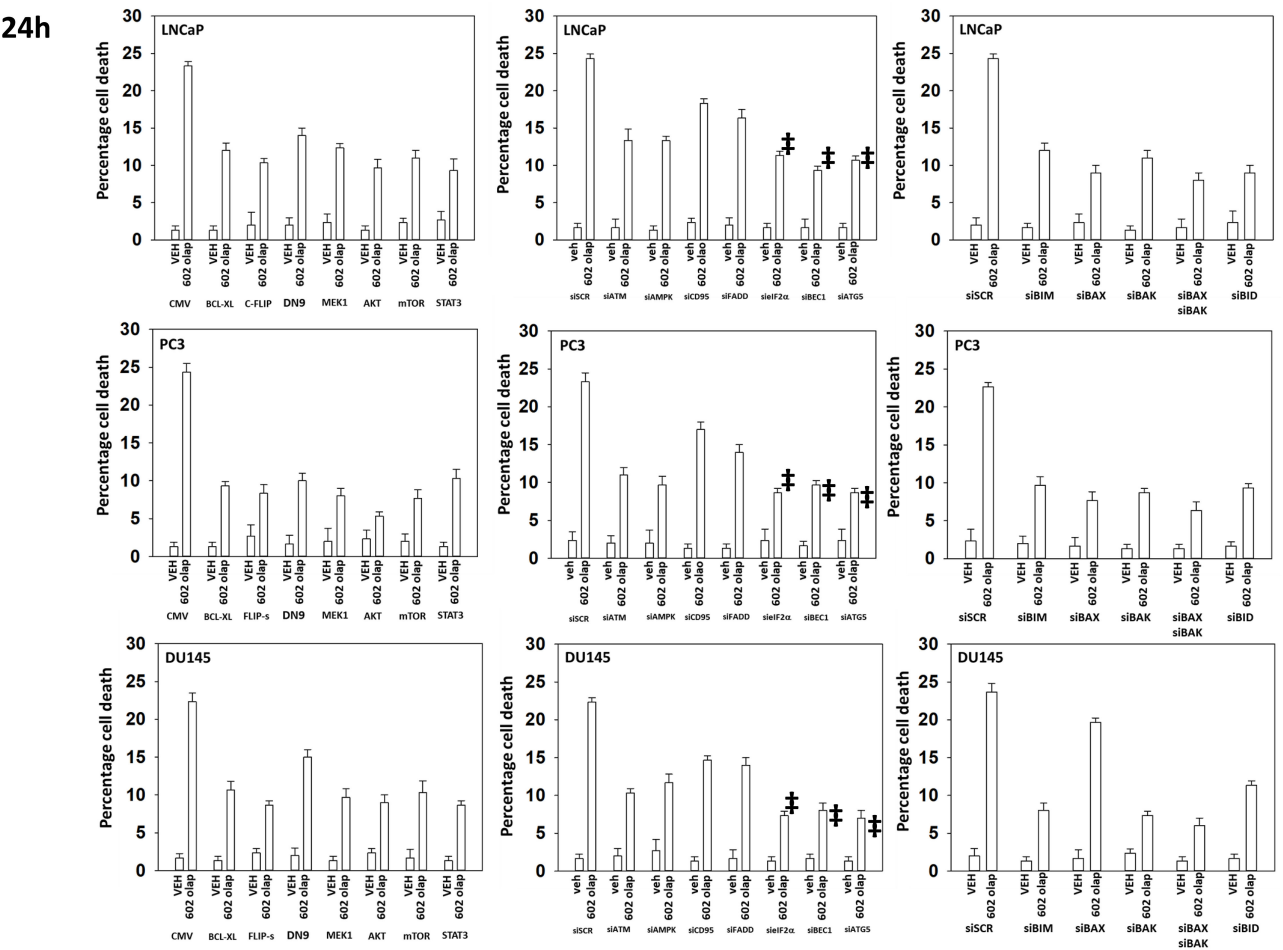
Knock down of Beclin1, ATG5 or eIF2α, or over-expression of FLIP-s significantly reduces the lethality of [GZ17-6.02 + olaparib]. Prostate cancer cells were transfected with an empty vector plasmid or with plasmids to express BCL-XL, FLIP-s, dominant negative caspase 9, activated MEK1, activated AKT, activated mTOR or activated STAT3. Cells were transfected with a scrambled siRNA or with siRNA molecules to knock down the expression of ATM, AMPKα, CD95, FADD, eIF2α, Beclin1, ATG5, BIM, BAX, BAK and BID. After 24h, cells were treated with vehicle control or with [GZ17-6.02 (2 μM) and olaparib (50 nM)] in combination for 24h. Cells were isolated, and viability determined by trypan blue exclusion. (n = 3 +/- SD). ‡p < 0.05 less than corresponding value in siATM or siAMPKα cells.

Additional studies then determined the relative importance of signaling proteins in tumor cell killing caused by the drug combination. Over-expression of BCL-XL or knock down of toxic BH3 domain proteins reduced killing, as did to a lesser extent knock down of CD95 or FADD (**Figure 11**). Over-expression of the caspase 8/10 inhibitor FLIP-s was significantly more protective than knock down of CD95 or FADD implying in our system caspase 8/10 was a component of an amplification loop in the cell killing process. Knock down of eIF2α, Beclin1 or ATG5 also reduced tumor cell death to a similar extent as did over-expression of FLIP-s whereas knock down of ATM less effective. Knock down of eIF2α was more cytoprotective than knock down of PKR (**Figures 6A**, **11**). Compiling data for the three lines, we observed in **Figure 5** that knock down of eIF2α reduced autophagosome formation by ~25% whereas in **Figure 11**, knock down of eIF2α reduced tumor cell killing by ~70%, as did knock down of Beclin1 or ATG5 (‡ p < 0.05).

**Figure 12:**
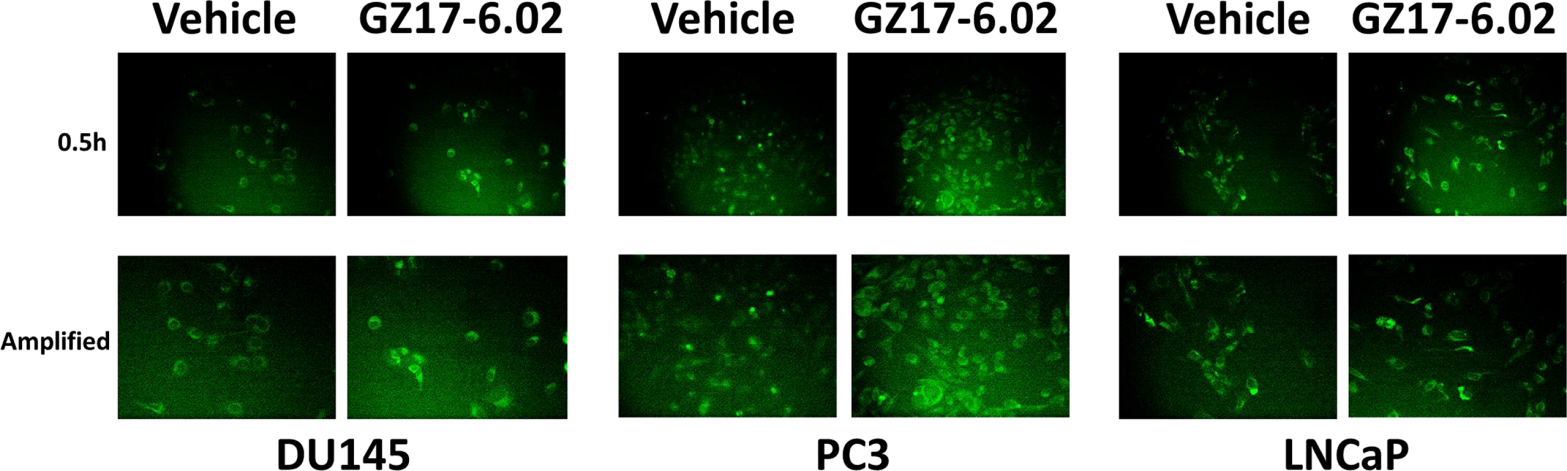
GZ17-6.02 rapidly activates ATM proteins localized around and outside of the nucleus. Cells were treated with vehicle control or with GZ17-6.02 (2 μM). Cells were fixed 0.5h and 1h after drug exposure. Cells were fixed in situ, permeabilized, stained with the indicated validated primary antibodies and imaged with secondary antibodies carrying red- and green-fluorescent tags. Total ATM protein levels and ATM localization within the cell did not alter over the time course (data not shown).

**Figure 13:**
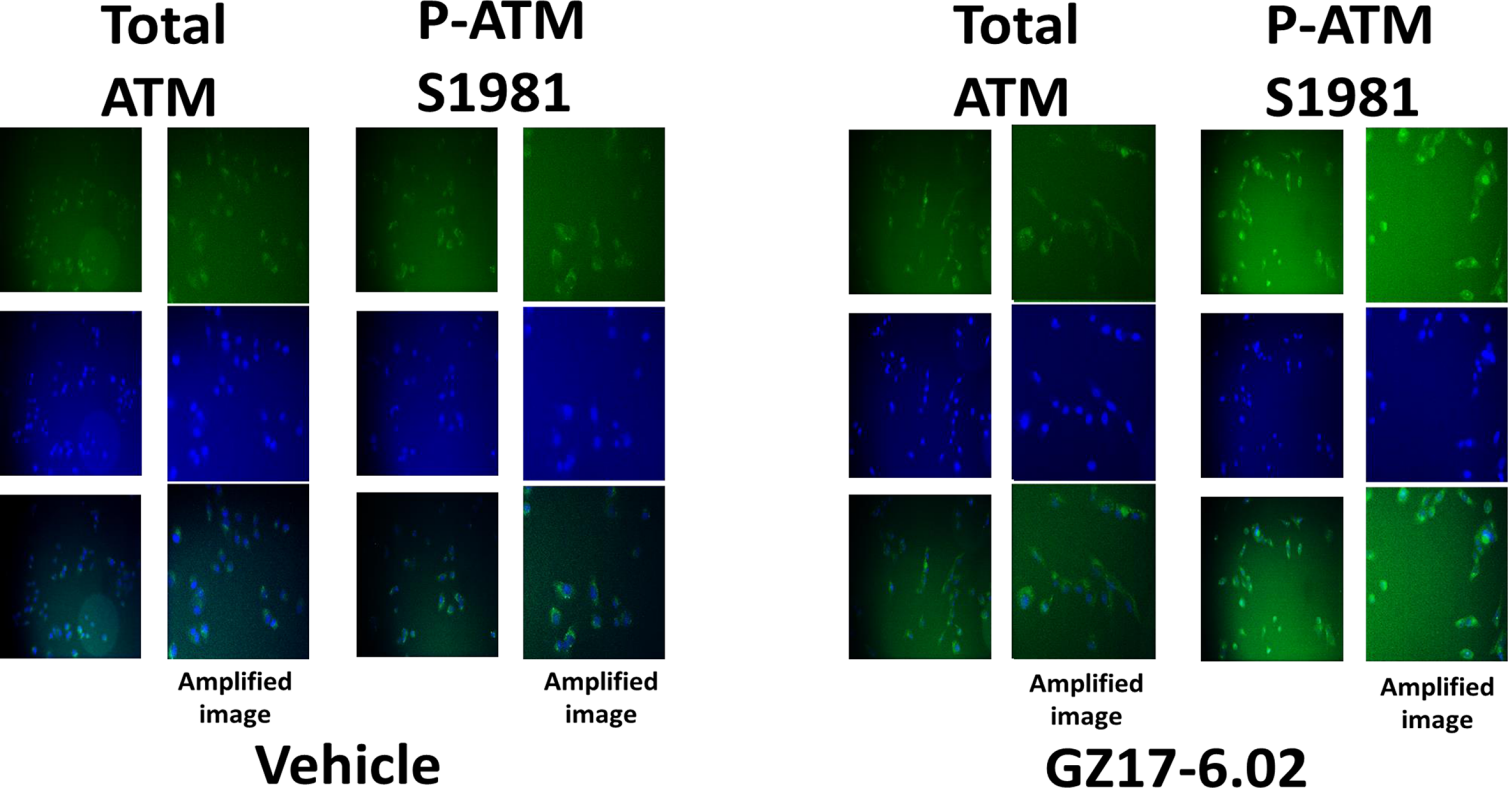
GZ17-6.02 rapidly activates ATM proteins localized around and outside of the nucleus. Cells were treated with vehicle control or with GZ17-6.02 (2 μM). Cells were fixed 6h after drug exposure. Cells were fixed in situ, permeabilized, stained with the indicated validated primary antibodies and imaged with secondary antibodies carrying red- and green-fluorescent tags. Cells were co-stained with DAPI. Total ATM protein levels and ATM localization within the cell did not alter over the time course (data not shown).

**Figure 14:**
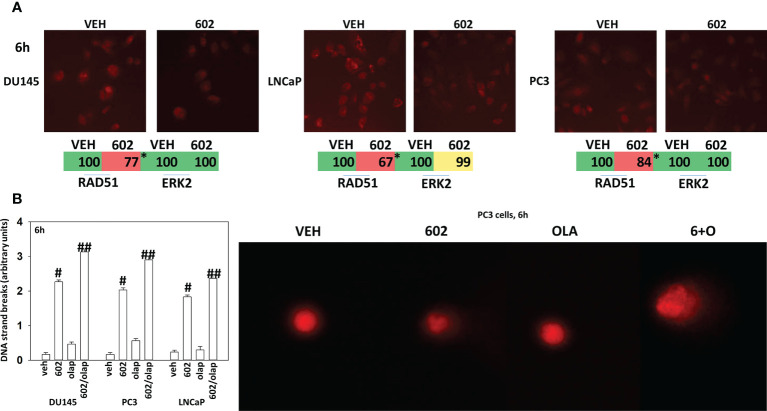
GZ17-6.02 reduces RAD51 expression and increases DNA damage. **(A)** Cells were treated with vehicle control or with GZ17-6.02 (2 μM). Cells were fixed 6h after drug exposure. Cells were fixed in situ, permeabilized, stained with the indicated validated primary antibodies and imaged with secondary antibodies carrying red- and green-fluorescent tags. The staining intensity of at least 100 cells per well/condition is determine in three separate studies. The data are the normalized amount of fluorescence set at 100% comparing intensity values for vehicle control (n = 3 +/-SD). #p < 0.05 greater than vehicle control; *p < 0.05 less than vehicle control. **(B)** Drug-treated cells, in suspension, were mixed with low melting point agarose and spread onto a microscope glass slide. After lysis of cells with detergent at a high salt concentration, DNA unwinding and electrophoresis was carried out at neutral pH (7-8). Tail moments were scored using a Method which classifies comets from grades 0–4 (11–13). One hundred comets were scored, and each comet assigned a value of 0 to 4 according to its class, the mean total score for the sample gel will be between 0 and 4 “arbitrary units.” (n = 3 +/-SD) # p < 0.05 greater than vehicle control; ##p < 0.05 greater than GZ17-6.02 alone value.

Based on the key role ATM signaling was playing in the regulation of ER stress signaling and tumor cell viability, we defined the localization of activated ATM, caused by exposure to GZ17-6.02. Within 30 min of exposure, the phosphorylation of ATM S1981 was increased (**Figure 12**). Increased staining was peri-nuclear. The total expression of ATM data did not change (not shown). We then examined the localization of phosphorylated ATM S1981 6h after treatment with GZ17-6.02. Although the majority of ATM protein was localized in the peri-nuclear region, phosphorylated ATM S1981 was localized in the nucleus (**Figure 13**). Within 6h of exposure, GZ17-6.02 had reduced the expression of RAD51 (**Figure 14A**). This data argues that the initial activation of ATM outside the nucleus reduces the expression of RAD51 which results in enhanced DNA damage in the nucleus and with activation of nuclear ATM. We then performed Comet assays to assess the amount of DNA damage being caused by the drugs alone or in combination. In comparison to GZ17-6.02, olaparib caused only modest levels of DNA damage (**Figure 14B**). The combination of GZ17-6.02 and olaparib caused greater amounts of DNA damage than treatment with GZ17-6.02 as a single agent.

**Figure 15:**
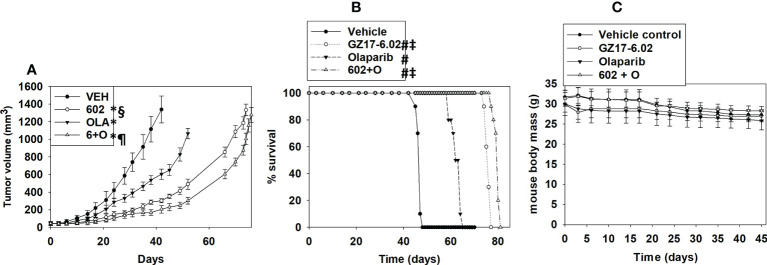
GZ17-6.02 profoundly suppresses the growth of AR+ prostate cancer tumors, prolonging survival, and interacts with olaparib to further reduce tumor growth. Male NRG mice supplied by the Massey Cancer Center Animal Core (∼20 g) were injected with 1.0×10^6^ male LNCaP cells into their rear flank (10 animals per treatment group). Tumors were permitted to form for 1 week with tumors at that time exhibiting a mean volume of approximately 50 mm^3^. Mice were treated by oral gavage once every day for 45 days with vehicle control, GZ17‐6.02 (50 mg/kg), olaparib (10 mg/kg) or the drugs in combination. Before, during and after drug treatment tumors were calipered every ~3 days as indicated in **(A)** and tumor volume was assessed up to 45 days later. Tumor volumes under each condition are plotted. * p < 0.05 reduced growth compared to vehicle control; § p < 0.05 reduced growth compared to olaparib single agent; p < 0.05 reduced growth compared to GZ17-6.02 single agent. Animals were humanely killed when the tumor volume reached approximately 2,000 mm^3^ due to ulceration. Animal survival was plotted on a Kaplan–Meier curve **(B)**. # p < 0.05 greater survival than vehicle control; ‡ p < 0.05 greater than olaparib single agent. In **(C)** the body mass of the mice in the study is presented over the 45-day treatment time course.

Finally, we determined whether GZ17-6.02 and olaparib interacted *in vivo* to suppress the growth of LNCaP tumors. As a single agent, GZ17-6.02 profoundly suppressed LNCaP tumor growth and prolonged animal survival (**Figures 15A, B**). As a single agent, olaparib more modestly reduced tumor growth, and also prolonged survival. The drugs in combination caused an additional smaller significant reduction in tumor growth that became particularly evident at ~days 35-40 and became even more obvious following discontinuation of drug treatment at day 45. However, there was no significant enhancement in survival comparing mice treated with GZ17-6.02 or with GZ17-6.02 and olaparib. Animal body mass under all treatment conditions did not significantly change over the 45-day time course (**Figure 15C**). Our data support the use of GZ17-6.02 as a therapeutic agent in patients with AR+ prostate cancer.

## Discussion

The present studies demonstrated that GZ17-6.02 interacted with the PARP1 inhibitor olaparib to kill prostate cancer cells. The lethal interaction between the drugs occurred regardless of whether the cells lacked expression of PTEN or had inactivating mutations in BRCA1/2. Multiple cell death effector pathways were engaged by the drug combination, in particular toxic autophagosome formation and death receptor signaling, both leading to mitochondrial dysfunction with mixed apoptotic and non-apoptotic killing. Upstream initiators of drug action were activation of ATM-AMPK signaling and increased endoplasmic reticulum stress signaling *via* PKR and eIF2α.

Compared to other solid tumor cell types we have previously tested, the activation of ATM caused by GZ17-6.02 was significantly greater in prostate cancer cells. And, furthermore, the activation of ATM was prolonged when compared to other tumor types. In HCT116 colon cancer cells we had previously found within 1h of GZ17-6.02 exposure that the expression of XPA and XPD had declined and the levels of RAD51 and RAD52 were enhanced (1). At this early timepoint, neither RAD51 nor RAD52 colocalized with nuclear P‐γH2AX staining, and instead, RAD51 and RAD52 were predominantly found in the perinuclear space. In support of those earlier observations, we discovered in prostate cancer cells that different pools of ATM protein were being activated at different time points. One hour following GZ17-6.02 treatment, ATM in the perinuclear space was activated whereas after 6h of exposure, ATM within the nucleus itself was phosphorylated. ATM outside of the nucleus has been argued to be regulated by reactive oxygen species however, our data in HCT116 cells demonstrated that ROS was not a component of GZ17-6.02 biology (6). In prostate cancer cells the expression of the DNA repair protein RAD51 was reduced by GZ17-6.02 after 6h of exposure and that DNA damage measured *via* comet assays persisted for up to at least 6h. The complicated molecular mechanisms by which GZ17-6.02 regulates ATM function outside and inside the nucleus, and DNA damage signaling, and repair will require studies beyond the present manuscript.

In other tumor cell types, GZ17-6.02 was shown to significantly activate PERK, which was responsible for drug-induced phosphorylation of serine 51 and inactivation of eIF2α. It was therefore unexpected when we discovered that in prostate cancer cells GZ17-6.02 did not significantly activate PERK, although the drug was still competent to significantly enhance eIF2α S51 phosphorylation. There are multiple kinases recognized to catalyze the phosphorylation of eIF2α S51 including PKR, GCN2 and HRI. Knock down of PKR, but not of PERK, GCN2 or HRI significantly reduced the ability of the drug combination to enhance S51 phosphorylation (14, 15).

Interferon-induced, double-stranded RNA-activated protein kinase (PKR), is a universally and constitutively expressed serine-threonine kinase with well-described roles in the regulation of immunity, metabolism, and neurological diseases (16–19). PKR signaling, *via* activation of the JNK MAPK pathway, has been linked to increased expression of inflammatory cytokines such as IL-1β and TNFα. Twenty years ago, we explored the Janus-faced nature of JNK signaling (20). In primary hepatocytes treated with a bile acid JNK signaling had the potential to both promote G1 progression and under different signaling circumstances, hepatocyte cell death. In prostate cancer cells, a similar situation has been reported. For example, inhibition of MEK4/7 and/or JNK1/2 reduced proliferation and invasion, presumably due to dephosphorylation of c-Jun and inactivation of AP-1 signaling complexes (21, 22). However, paclitaxel toxicity in prostate cancer cells requires activation of JNK (23). Unlike in other types of tumor cell, GZ17-6.02 and olaparib caused a robust activation of JNK1/2 and knock down of PKR prevented this response (1). PKR can activate the transcription factor NFκB (18). In other tumor types, we observed either no effect or inactivation of NFκB signaling caused by GZ17-6.02 whereas in prostate cancer cells, the drugs in combination activated NFκB. Activation of NFκB signaling in prostate cancer is most often linked to increased proliferation and tumor development (24, 25). However, expression of a constitutively active form of p65 NFκB causes apoptosis in prostate cancer cells, in part due to increased expression of the death receptor CD95 (FAS) and enhanced levels of toxic BH3 domain proteins (26). Future studies will be required to link signaling by PKR, through to JNK and NFκB signaling, and to the inflammatory microenvironment in prostate cancer tumors treated with GZ17-6.02 and olaparib.

One of the first manuscripts published examining the biology of GZ17-6.02 linked its effects on pancreatic cancer cells to the regulation of super-enhancers (SEs) (27). SEs are specialized areas of the genome which drive high rates of transcription and play a key role in cell biology. Using ChIP-Seq, it was discovered that GZ17-6.02 altered the acetylation of the genes, lowered the activities of major transcription factors and stem cell markers. GZ17-6.02 reduced both Oct-4 expression and decreased in the occupancy of OCT-4 in the entire genome. In prostate cancer, SEs force the tumor cells to become addicted to dysregulated transcription programs mediated by proteins such as BRD4, CDK7, and ERG (28–30). Whether GZ17-6.02 can regulate SEs in prostate cancer, and link altered transcription to the biology described herein will require studies beyond the scope of this paper.

There remains in the cancer therapeutics field a controversy as to the relevance of autophagy either in reducing the efficacy of therapeutic regimens or playing an active role in the tumor cell killing process. We discovered that knock down of eIF2α was more cytoprotective than knock down of PKR, suggesting that PKR is not the only eIF2α kinase playing a regulatory role in our system. Compiling and comparing the data from the three lines we examined, we determined that knock down of eIF2α reduced autophagosome formation by only ~25% whereas knock down of eIF2α reduced tumor cell killing to a significantly greater extent, ~70%. In comparison, knock down of either Beclin1 or ATG5 reduced both autophagy and cell killing by ~70%. Thus, inhibition of autophagy by only ~25% is sufficient to reduce killing by ~70%. This suggests that above a certain threshold, estimated to be ~75% of the total expected autophagosome formation, the GZ17.602 olaparib drug combination causes autophagy to become toxic. And, to achieve the higher toxic level of autophagosome formation and autophagic flux, ER stress signaling, i.e., eIF2α inactivation, must be intact.

Although we do not know how much inhibition of PARP is caused by 10 mg/kg in a mouse tumor, prior studies in patients have been performed. With a 400 mg BID olaparib dosing schedule, pharmacokinetic (PK) investigations demonstrated a rapid absorption of olaparib (peak plasma concentration was between 1 and 3 h after dosing) and elimination (terminal-elimination with a half-life of ~5 to ~7 h) of olaparib (31, 32). Pharmacodynamic (PD) assessments demonstrated PARP inhibition in surrogate samples; peripheral blood mononuclear cells (PBMCs), and tumor tissue. These studies revealed that PARP inhibition in PBMCs rapidly reached a plateau of approximately sixty percent.

GZ17-6.02 is undergoing phase I evaluation in cancer patients. Hence, in addition to our *in vitro* mechanistic analyses, we performed *in vivo* studies to define the *in vivo* interaction between GZ17-6.02 and olaparib. Our *in vivo* data argue that GZ17-6.02 may have single agent potential to suppress the growth of AR+ prostate cancer tumors and prolong survival. Although GZ17-6.02 and olaparib interacted to suppress growth below that of GZ17-6.02 as a single agent, in the absence of drugs, the tumors regrew to such an extent that there was no difference in animal survival. Whether prolonged (> 45 days) exposure of the tumors to GZ17-6.02 and olaparib would ultimately result in a significant enhancement in survival is not known.

## Data availability statement

The raw data supporting the conclusions of this article will be made available by the authors, without undue reservation.

## Ethics statement

The animal study was reviewed and approved by Studies were performed according to the U.S. Department of Agriculture regulations under the VCU IACUC protocol AD20008.

## Author contributions

LB and JR performed the studies. PD directed the studies. CW collaborated with PD to develop the studies and critically read the final manuscript. All authors contributed to the article and approved the submitted version.

## Funding

Support for the present study was funded from philanthropic funding from Massey Cancer Center and the Universal Inc. Chair in Signal Transduction Research. PD acknowledges funding Genzada Pharmaceuticals USA, Inc. The funder was not involved in the study design, collection, analysis, interpretation of data, the writing of this article or the decision to submit it for publication.

## Acknowledgments

Additional support for the present study was funded from philanthropic funding from Massey Cancer Center and the Universal Inc. Chair in Signal Transduction Research. Services in support of the research project were provided by the VCU Massey Cancer Center Mouse Core, supported, in part, with funding from NIH-NCI Cancer Center Support Grant P30 CA016059. We thank Dr. Roy Sabo (VCU, Biostatistics), for biostatistical assistance during these studies. PD and CW thank Dr. Daniel Von Hoff for expert guidance during performance of these studies and for editing the manuscript.

## Conflict of interest

Author CW is a paid officer at Genzada Pharmaceuticals. PD is a Consultant and Key Scientific advisor to Genzada Pharmaceuticals.

The remaining authors declare that the research was conducted in the absence of any commercial or financial relationships that could be construed as a potential conflict of interest.

## Publisher's note

All claims expressed in this article are solely those of the authors and do not necessarily represent those of their affiliated organizations, or those of the publisher, the editors and the reviewers. Any product that may be evaluated in this article, or claim that may be made by its manufacturer, is not guaranteed or endorsed by the publisher.

## Abbreviations

ER, endoplasmic reticulum; AMPK, AMP-dependent protein kinase; mTOR, mammalian target of rapamycin; JAK, Janus kinase; STAT, Signal Transducers and Activators of Transcription; MAPK, mitogen activated protein kinase; CMV, empty vector plasmid; si, small interfering; SCR, scrambled; VEH, vehicle; 602, GZ17-6.02; Olap, olaparib; ER stress, GZ17-6.02, olaparib.
